# Bacterial Diversity in Deep‐Sea Sediments of the North Atlantic Ocean and Their Biosynthesis of Secondary Metabolites

**DOI:** 10.1111/1758-2229.70092

**Published:** 2025-07-09

**Authors:** Pietro Marchese, Joe Bracegirdle, Ryan Young, Emanuele Ferrari, Laura Garzoli, J. Mary Murphy, Maria Tuohy, A. Louise Allcock, Bill J. Baker

**Affiliations:** ^1^ Molecular Ecology Group Water Research Institute, Italian National Research Council Verbania Italy; ^2^ Department of Chemistry University of South Florida Tampa Florida USA; ^3^ Molecular Glycobiotechnology, School of Natural Sciences University of Galway Galway Ireland; ^4^ Regenerative Medicine Institute, School of Medicine University of Galway Galway Ireland; ^5^ Martin Ryan Institute, School of Natural Sciences University of Galway Galway Ireland

**Keywords:** antibiotics, marine, metabolomics, molecular networking, phylogenesis, prokaryotes

## Abstract

Oceanic bathyal benthos harbours extremotolerant microbial species living with low nutrient availability, high hydrostatic pressure, and low temperature. Within their community, bacteria can secrete signalling molecules to gain a competitive advantage over space and nutrients, often representing bioactive compounds with relevant biotechnological potential. In this study, we investigate the species diversity of culturable bacteria associated with sediments sampled at the deepest margin of the European Western Continental Shelf. Furthermore, we explore their biosynthesis of known and new bioactive molecules. We isolated 144 bacterial strains belonging to 60 different taxa, including potentially new species, two species never recorded in the marine environment, and 15 new to the deep‐sea. Investigation of the bacteria ability to synthesise ecologically relevant molecules was performed through genetic screening and metabolomics. Our results highlight a high rate of antibiotic‐producing bacteria as well as the biosynthesis of potentially quorum‐sensing compounds. Actinobacteria displayed the highest potential to biosynthesise bioactive molecules such as polyketides and non‐ribosomal peptides, with a high rate of isolates carrying biosynthetic genes as well as the highest number of molecules detected by mass spectrometry. In this study, the deep‐sea demonstrates a valuable source of unknown microbial and chemical diversity, supporting initiatives aimed at its protection from anthropogenic disturbance.

## Graphical abstract

In this study, we isolated culturable bacteria associated with oceanic sediments and studied their production of secondary metabolites using metabolomics and genetic screening. Bacteria were cultivated from depths as great as 2000 m below sea level, with the majority of culturable taxa from all sampling sites belonging to the Pseudomonadota phylum. Our findings indicate that deep‐sea Actinomycetes and Pseudomonadota biosynthesise a wider range of polyketides than non‐ribosomal peptides, whereas Firmicutes showed the opposite trend.

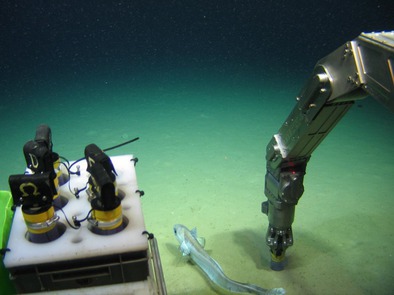

## 1 Introduction

Bacteria are major constituents of global ecosystems, contributing to 15% of the World's total biomass and performing key ecological roles in aquatic and terrestrial habitats (Mendes et al. 2013; Schimel and Schaeffer 2012). In the marine environment, bacteria are present in pelagic microbial communities at depths ranging from surface water to the abyss, as well as in benthic sediments at all depths. Bacterial communities constitute up to 25% of the euphotic zone total biomass and establish symbiotic relationships with photoautotrophic microalgae directly influencing their productivity (Amin et al. 2015; Cole et al. 1988). They have major roles in cycling organic carbon from primary producers (Cole et al. 1988; Ducklow 2006) as well as in nutrient transfer across the water column by forming marine snow particulate organic matter sinking to abyssopelagic habitats (Sanz‐Sáez et al. 2023). Marine snow brings nutrients and microorganisms to the deep‐sea, the largest environment existing on Earth, typically characterised by extreme conditions such as high hydrostatic pressure, absence of light and low temperature. Despite extreme environmental conditions often characterised by low nutrient availability, the biodiversity reported from deep‐sea habitats is rich and comparable to that found in tropical rainforests and coral reefs (Appeltans et al. 2012.  Grassle and Maciolekt 1992).

At the bottom of the ocean, sediment‐associated bacterial communities generate assemblages influenced by both biotic and abiotic factors. Several studies have indicated organic carbon content and organic carbon source chemical composition as major drivers of biodiversity (Bienhold et al. 2016; Goffredi and Orphan 2010; Hoffmann et al. 2017), while others highlighted depth and geomorphology. For example, the transition between upper and lower continental slope (~1000 mbsl) is characterised by sharp changes in sea bottom and water column conditions (Costello et al. 2017; Watling et al. 2013), and is a stronger driver of biodiversity compared to geographic location, water temperature and sediment depth, as indicated by Trouche et al. (2021). Bacterial diversity associated with oceanic benthic sediments was studied in the past using both culture‐dependent and culture‐independent methods, highlighting similar biogeographical diversity patterns. The dominant phylum in marine sediments across all oceans is Pseudomonadota, while the phylum Actinobacteria is significantly represented at most investigated sites. A meta‐analysis on 68 deep‐sea sediments from the International Census of Marine Microbes showed worldwide dominance of Gammaproteobacteria (~25% of overall microbial community), followed by Deltaproteobacteria (~15%), Alphaproteobacteria (~10%) and Actinobacteria (< 10%), and low abundance of Bacteroidetes and Firmicutes (< 5%) (Zinger et al. 2011). Similarly, a more recent worldwide study (Bienhold et al. 2016) highlighted dominance of Gammaproteobacteria (20%), followed by Alphaproteobacteria and Actinobacteria (> 12%), Deltaproteobacteria and Betaproteobacteria (> 8%), and lower abundance of Bacteroidetes and Firmicutes (3%). Sediment bacterial diversity in the Atlantic Ocean was investigated in several studies implementing metabarcoding with Next Generation Sequencing technology (Bienhold et al. 2016; Cerqueira et al. 2015; Reese et al. 2018; Trouche et al. 2021), which highlighted the phylum Pseudomonadota as dominant and abundances of Gamma‐ and Alpha‐proteobacteria classes in ranges comparable to global reports.

Bacterial diversity recorded by culture‐dependent methods in sediments from the Pacific Ocean highlighted the dominance of Gammaproteobacteria (75%), while a minority of Firmicutes (10%), Alphaproteobacteria (7%), Bacteroidetes (7%) and Actinobacteria (1%) were detected (Dang 2009). In this study, *Halomonas* and *Pseudomonas* were the most diverse genera isolated, followed by *Bacillus* and *Sporosarcina*. Further studies from the Pacific Ocean (Romanenko 2013) reported the dominance of cultivated Firmicutes (27%), followed by Actinobacteria (21%), Gammaproteobacteria (20%), Alphaproteobacteria (18%), and Bacteroidetes (14%). The most diverse genera were *Bacillus*, followed by *Sulfitobacter*, *Paenibacillus*, *Paenisporosarcina*, and *Nocardiopsis*. In the Atlantic Ocean, a previous study from sediment samples in the southeast (da Silva et al. 2013) showed a community mainly composed of Gammaproteobacteria (55%), Firmicutes (38.5%), and Actinobacteria (5.5%), with genera diversity being dominated by *Halomonas*, *Bacillus*, Staphylococcus, and *Psychrobacter*. Culturable bacteria diversity observed in Caribbean Sea sediments was dominated by Firmicutes (51%), followed by Pseudomonadota (34%) and Actinobacteria (15%), with dominant genera being *Bacillus, Fictibacillus, Halomonas*, and *Pseudomonas* (Blandon et al. 2022). Bacterial diversity recorded in the bathypelagic realm of oceans worldwide was isolated by Sanz‐Sáez et al. (2023), indicating the dominance of the genera *Sulfitobacter*, *Halomonas*, and *Erythrobacter*, highlighting these lineages as potentially key bacteria involved in cycling organic carbon from shallow water to the sea bottom as well as in the benthic microbial community.

Bacterial success in the colonisation of their habitat is often mediated by secretion of secondary metabolites with signalling function (Tyc et al. 2017). Quorum sensing (QS) is the bacterial system for intra‐ and inter‐species communication, which is active in both soil and aquatic communities (Waters and Bassler 2005). Largely characterised mediators of this function are acyl homoserine lactones. Gamma‐butyrolactones and other lactones have also been demonstrated to influence microbially driven biogeochemical cycles, symbiosis, or community structure (Hmelo 2017; Hmelo et al. 2011; Martin et al. 2017; Romero et al. 2011). Other signaling molecules are antibiotics, which secreted at sub‐inhibitory levels also mediate cell‐to‐cell communication. For example, beta‐lactams and aminoglycosides improved biofilm formation of *Pseudomonas aeruginosa* (Bagge et al. 2004; Hoffmann et al. 2017), while membrane pore‐forming depsipeptides or polyketides induce signalling cascades for biofilm modulation in response to cytoplasmic potassium leakage (López et al. 2008). Lipopeptides, a class of molecules including the typically marine and microbial depsipeptides (Hamada and Shioiri 2005), show surfactant, antimicrobial, and cytotoxic functions (Gutiérrez‐Chávez et al. 2021). Polyketides, a structurally diverse group of molecules with complex biosynthetic pathways, are often associated with biological activities such as antibiotic, insecticidal, or cytotoxicity (Kirst 2010; Schneemann et al. 2010; Walsh 2003). Additional bioactive natural products, such as the plant and animal bioactive molecule rapamycin, are formed by combination of polyketide and peptide moieties to form hybrid polyketide‐non ribosomal peptide compounds (Abraham and Wiederrecht 1996; Xiong and Sheen 2012). Terpenes are often volatile molecules mainly studied in plants but widely distributed in bacteria (Yamada 2015), with demonstrated antibiotic and cytotoxic activities (Gordaliza 2010; Takahashi et al. 1983), as well as allelopathic function in marine macrophytes (Rasher et al. 2011). Major efforts were implemented to study interspecies interaction driven by natural products within soil rather than aquatic microbial communities, but sporadic evidence demonstrated chemical signals‐driven communication also in aquatic bacteria (Amin et al. 2015; Fei et al. 2020; Pérez‐Rodríguez et al. 2015). The ability to communicate through chemical signalling is encoded within genomic Biosynthetic Gene Clusters (BGCs), sequences often conserved by organisms across variable environments (Yi et al. 2024) which can be mapped in microbial genomes by simple PCR screening (Ayuso et al. 2005; Ayuso‐Sacido and Genilloud 2005). Moreover, the rapid detection of known bioactive compounds and their unknown analogues is facilitated by the recent curation of metabolomic repositories containing mass spectrometry fragmentation patterns of millions of molecules, such as the Global Natural Products Social (GNPS) database (Wang et al. 2016). Such technology is rapidly advancing the study of microbial natural products providing a platform to rapidly annotate molecules contained in biological samples, and will boost our understanding of microbial metabolites' ecological role in the natural environment (Millán‐Aguiñaga et al. 2019).

In this study, we implemented a culture‐dependent strategy to investigate the bacterial diversity associated with deep‐sea sediments sampled along the European continental slope, off the coast of Ireland. Microbial taxonomy aimed at species‐level identification to advance our understanding of bacterial biogeographic distribution, providing relevant data from the deep‐sea sediments of a previously unexplored geographical sector. Moreover, the isolated bacteria offered valuable strains to explore the biosynthetic capabilities of extremotolerant bacteria. We performed genetic screening and metabolomic characterisation of deep‐sea bacteria to evaluate their production of bioactive compounds, which are known for influencing community structure and hold potential for biotechnological applications. To the best of our knowledge, this study represents the first report on deep‐sea bacterial diversity from sediments of this geographical area, as well as the first report on the ability of deep‐sea sediment‐inhabiting bacteria to synthesise bioactive compounds potentially driving community structure in this extreme and under‐characterised environment.

## 2 Results

### 2.1 Deep‐Sea Bacteria Diversity

**FIGURE 1:**
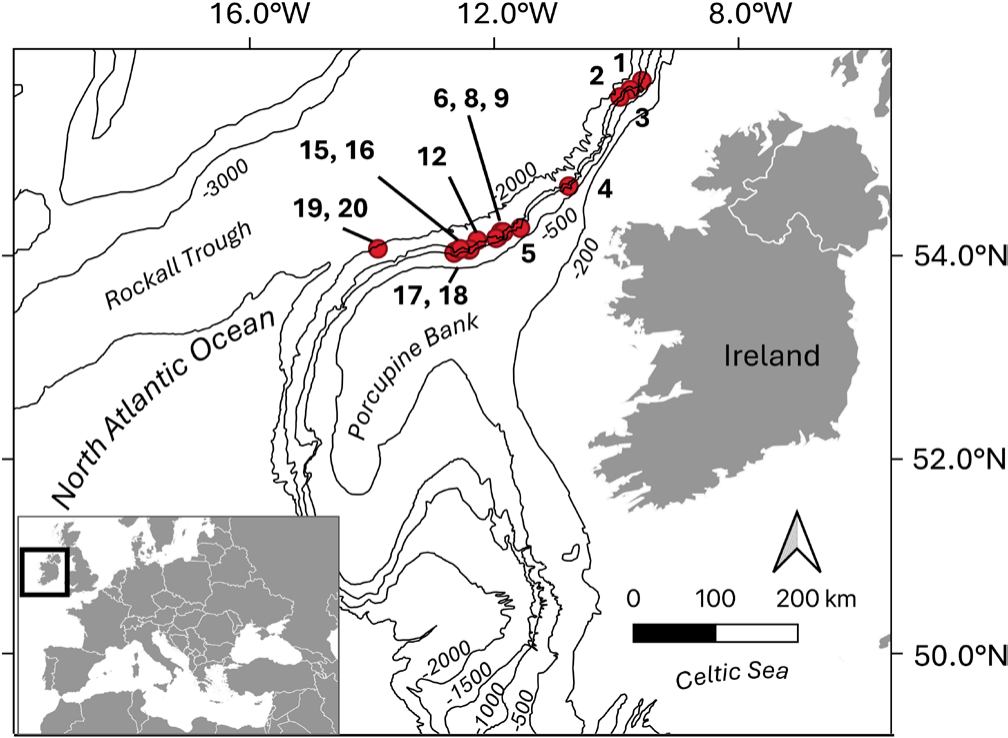
Map of the sampling area. Sediments were collected along the deep edge of the Porcupine Bank at 15 sites (Sediment 1–20) at depths ranging from 933 to 2150 mbsl.

A sampling campaign conducted over the continental slope declining from the Porcupine Bank, off the west coast of Ireland (Figure 1), provided 15 sediment cores for microbial isolation (numbered non‐continually from 1 to 20, Table 1). A total of 144 bacterial morphotypes representing different strains were isolated in pure culture and taxonomically assigned. Full‐length 16 s rDNA gene sequencing and nucleotide‐BLAST alignment allowed the identification of 60 taxa, of which 45 were identified at the species level and 15 at the genus level. Phylogenesis was used to confirm bacterial taxonomy and evaluate the presence of new species. Most isolates (140 out of 144) showed sequence similarity higher than 99% with previously deposited sequences in GenBank. Two isolates showed a similarity of 98% and two isolates a similarity of 97%, potentially representing new species affiliated with the *Psychrobacter* and *Marinobacter* genera. Moreover, a subset of isolates with high sequence similarity to previously deposited sequences of uncultured Arctic bacteria were assigned to the *Erythrobacter* genus, closely related to *E. citreus* . Phylogenetic trees supporting taxonomy assignments are reported in Figures S1–S13.

**TABLE 1:** Sediment samples from the deep North Atlantic Ocean processed for bacteria isolation, location, and biodiversity indexes.

Sample	Substrate	Latitude	Longitude	Depth (−m)	Temp. (°C)	Salinity (PSU)	Biodiversity indexes^a^			
							N° isolates	*H′*	1‐Lambda	*J'*
S1	Sandy mud	55°38′54.4″N	9°35′02.2″W	955	7.0	35.2	16	0.727	1.575	0.690
S2	Sandy mud	55°38′55.9″N	9°46′36.0″W	933	6.8	35.2	2	0.5	0.693	1
S3	Sandy mud	55°29′51.0″N	9°56′31.0″W	1567	4.0	34.9	7	0.776	1.55	0.942
S4	Sandy mud	54°39′47.2″N	10°46′40.4″W	1317	4.5	35.0	2	0.5	0.693	1
S5	Sand	54°15′55.8″N	11°34′18.0″W	983	7.1	35.2	7	0.449	0.796	0.739
S6	Copepod shells+ sandy mud	54°13′35.6″N	11°52′50.8″W	1420	4.2	35.0	1	0	0	1
S8	Muddy sand	54°10′48.0″N	11°56′21.9″W	1598	3.9	34.9	3	0.444	0.637	0.945
S9	Muddy sand	54°10′32.3″N	11°57′05.9″W	1345	4.4	35.0	7	0.694	1.277	0.897
S12	Muddy sand	54°03′46.2″N	12°23′49.8″W	1287	5.3	35.0	1	0	0	1
S15	Muddy sand	54°03′42.5″N	12°32′58.3″W	1289	5.6	35.1	2	0.5	0.693	1
S16	Sandy mud	54°03′41.0″N	12°33′00.4″W	1337	5.7	35.1	2	0.5	0.693	1
S17	Muddy sand	54°01′34.9″N	12°38′37.4″W	1648	4.1	35.0	57	0.929	2.927	0.747
S18	Sandy mud	54°01′39.4″N	12°39′24.9″W	1311	5.3	35.0	6	0.778	1.561	0.952
S19	Sandy mud	54°04′01.1″N	13°54′14.6″W	2058	3.4	34.9	20	0.925	2.692	0.923
S20	Muddy sand	54°04′11.8″N	13°54′18.0″W	2150	3.4	34.9	11	0.876	2.146	0.950

*Note:* Samples were collected during cruise CE18012 onboard the R.V. Celtic Explorer. Sediment composition was analysed using laser particle sizing; geodetic coordinates are indicated in WGS 84 Web Mercator; depth, temperature and salinity data were recorded using a CTD profiler mounted on ROV Holland I used for sampling.

^a^ Biodiversity indexes within sampling sites: Shannon‐Weaver index (H′), Gini‐Simpson index (1‐Lambda) and Pielou's evenness (J').

**FIGURE 2:**
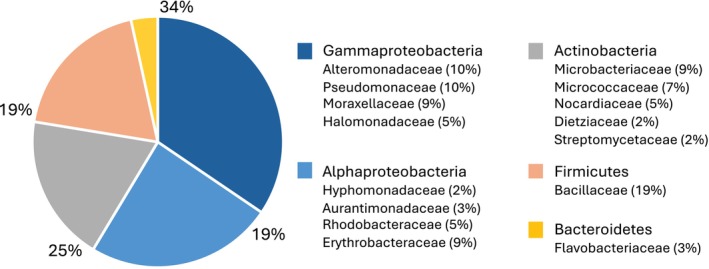
Bacteria diversity retrieved from deep‐sea sediments. Overall diversity encompassed 5 phyla and 15 families. Proportions are expressed as a percentage of taxa belonging to each lineage compared to the total number of cultivated bacteria.

Cultivable bacterial morphotypes (strains) isolated from each sediment core ranged from one to 26, with most cores carrying two to seven. We identified bacteria belonging to five phyla, nine orders, 15 families, and 27 genera (Figure 2). Pseudomonadota were the most widespread: they were isolated from seven out of 15 sediments and represented 53% of all isolated strains. The majority of Pseudomonadota isolates belonged to the class Gamma‐proteobacteria, representing 34% of retrieved taxa, followed by Alpha‐proteobacteria, which represented 19% of the isolates. Actinobacteria and Firmicutes were isolated with a smaller proportion (25% and 19%, respectively), but both were retrieved from 11 out of 15 sediments. A smaller proportion of isolates was represented by Bacteroidetes (3%, 2 sediments).

**FIGURE 3:**
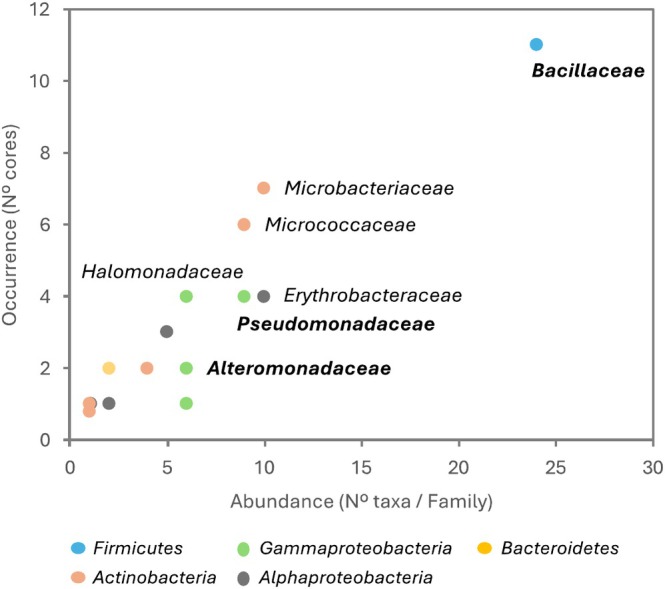
Abundance versus occurrence of families isolated from deep‐sea sediments. Family names are reported for lineages retrieved from > 25% of samples. In bold are reported families that showed higher diversity compared to the rest of the isolates (> 6 taxa per family).

Biogeographic evaluation of the cultured community was performed to visualise the abundance of bacterial lineages on the different sediment cores (Figure 3). Results highlighted the family *Bacillaceae* as the most recurrent, isolated from over 70% of investigated cores but not found at depths greater than 1648 mbsl. The following most recurrent families were *Microbacteriaceae* and *Micrococcaceae*, detected from over 40% of processed cores. Erythrobacteraceae, *Pseudomonadaceae*, and *Halomonadaceae* were obtained from 27% of cores, *Rhodobacteraceae* from 20%; and *Nocardiaceae*, *Flavobacteriaceae*, *Aurantimonadaceae*, *Dietziaceae*, *Hyphomonadaceae*, and *Streptomycetaceae* were only retrieved from 3% or fewer cores. Three widely distributed families also showed higher diversity compared to the rest of the dataset: *Bacillaceae* (13 taxa from 24 isolates), Pseudomonadaceae (6 taxa from 9 isolates), and *Alteromonadaceae* (5 taxa from 6 isolates).

The most widespread bacterial genus observed across sediment cores was *Bacillus* (8 taxa), followed by *Pseudomonas* (6 taxa), *Psychrobacter* (5 taxa), and *Erythrobacter* (5 taxa). *Bacillus licheniformis* was the species most frequently isolated, colonising five out of fifteen cores, while *Bacillus firmus* , *Halomonas meridiana* , *Micrococcus luteus*, and *Microbacterium* sp. were retrieved from a maximum of three cores. Two taxa isolated in this study were detected for the first time in the marine environment: *Lysinibacillus telephonicus* and *Glutamicibacter protophormiae*. Fifteen taxa were recorded for the first time in a deep‐sea environment (Table 2). For bacterial taxonomy, 144 new sequences of bacterial genomic DNA (16S rRNA gene) were generated and deposited in the NCBI database (Table S2).

**TABLE 2:** Marine bacterial taxa isolated from deep‐sea sediments and recovered from other marine substrates worldwide.

	Taxa	Substrate	N° Isolates	From deep sea	From Atlantic Ocean	From marine environment worldwide
Actinobacteria	*Citricoccus parietis*	S17, S19	2	[1]	FR	[2]
	*Dietzia maris*	S8	2	[3]	[3]	—
	*Glutamicibacter protophormiae*	S3, S19	2	FR	FR	FR
	*Kocuria rhizophila*	S1, S3	2	[4]	[4]	—
	*Leucobacter* sp.	S5	1	[5]	[5]	—
	*Microbacterium oxydans*	S5	5	[6]	FR	[7]
	*Microbacterium profundi*	S9, S19	2	[8]	FR	—
	*Microbacterium* sp.	S2, S17, S19	6	[9]	[9]	—
	*Micrococcus luteus*	S1, S8, S9	3	[10]	[10]	—
	*Nocardioides* sp.	S20	1	[11]	[11]	—
	*Rhodococcus fascians*	S17	4	[12]	[12]	—
	*Rhodococcus* sp.	S17	1	[12]	[12]	—
	*Salinibacterium amurskyense*	S17, S18, S20	3	[13]	[13]	[14]
	*Streptomyces violascens*	S15	1	[15]	FR	—
Alphaproteobacteria	*Aurantimonas coralicida*	S17	2	FR	FR	[16]
	*Aurantimonas* sp.	S17	1	[17]	[17]	—
	*Erythrobacter citreus*	S17	7	[18]	[18]	—
	*Erythrobacter pelagi*	S20	1	[18]	[18]	—
	*Erythrobacter seohaensis*	S17, S19	3	FR	FR	[19]
	*Erythrobacter* sp.1	S17, S18	2	[18]	[18]	—
	*Erythrobacter* sp.2	S17	9	[18]	[18]	—
	*Hyphomonas* sp.	S17	1	[20]	[20]	—
	*Limimaricola cinnabarinus*	S17	1	[21]	FR	—
	*Sulfitobacter pontiacus*	S19, S20	4	[22]	FR	—
	*Sulfitobacter pseudonitzschiae*	S15	2	FR	[23]	[23]
Gammaproteobacteria	*Alteromonas naphthalenivorans*	S19	1	[24]	[24]	[25]
	*Alteromonas* sp.	S19	2	[26]	[27]	[28]
	*Alteromonas undina*	S20	1	FR	FR	[29]
	*Halomonas meridiana*	S1, S9, S20	10	[30]	[30]	—
	*Halomonas titanicae*	S20	2	[31]	[31]	—
	*Halomonas venusta*	S17, S20	7	[32]	[33]	—
	*Marinobacter* sp.	S19	1	[18]	[18]	—

	*Pseudoalteromonas* sp.	S19	1	[34]	[34]	—
	*Pseudomonas abyssi*	S17	2	[58]	FR	—
	*Pseudomonas marincola*	S19	1	[35]	FR	—
	*Pseudomonas protegens*	S5	1	FR	FR	[36]
	*Pseudomonas* sp.1	S19	2	[37]	[37]	—
	*Pseudomonas* sp.2	S19, S20	3	[37]	[37]	—
	*Psychrobacter celer*	S17	1	FR	[38]	[39]
	*Psychrobacter nivimaris*	S17	1	[40]	[41]	—
	*Psychrobacter okhotskensis*	S17	1	FR	FR	[42]
	*Psychrobacter pulmonis*	S17	1	FR	FR	[43]
	*Psychrobacter* sp.	S17	2	[18]	[18]	—
*Stutzerimonas zhaodongensis*	S17, S19	3	[44]	[44]	—
Firmicutes	*Bacillus firmus*	S4, S6, S16	3	[45]	[46]	—
	*Bacillus licheniformis*	S1, S3, S4, S66, S18	9	[45]	[47]	—
	*Bacillus mycoides*	S1	1	FR	[48]	—
	*Bacillus niabensis*	S3, S17	3	[49]	FR	[50]
	*Bacillus paralicheniformis*	S1, S18	3	[51]	FR	—
	*Bacillus pumilus*	S1, S17	3	[52]	[52]	[53]
	*Bacillus* sp.1	S18	1	[52]	[52]	—
	*Bacillus* sp.2	S3, S15	3	[52]	[52]	—
	*Lysinibacillus telephonicus*	S12	1	FR	FR	FR
	*Paenibacillus amylolyticus*	S2	1	[45]	FR	—
	*Peribacillus simplex*	S15	1	[53]	FR	—
	*Pseudalkalibacillus hwajinpoensis*	S3	1	FR	FR	[54]
	*Virgibacillus salarius*	S9	3	FR	FR	[55]
Bacteroidetes						
	*Gramella marina*	S19	1	FR	FR	[56]
	*Leeuwenhoekiella aequorea*	S17	1	FR	FR	[57]

*Note:* Reference: [1] (Ettoumi et al. 2016), [2] (Kalinovskaya et al. 2011), [3] (Gao et al. 2015), [4] (Williams et al. 2020), [5] (Ribeiro et al. 2023), [6] (Sfanos et al. 2005), [7] (Kim et al. 2013), [8] (Wu et al. 2013), [9] (Tomasino et al. 2021), [10] (Uniacke‐Lowe et al. 2023), [11] (Bertrand et al. 2013), [12] (Stach et al. 2003), [13] (Hirayama et al. 2015), [14] (Han et al. 2003), [15] (Viegelmann et al. 2014), [16] (Denner et al. 2003), [17] (He et al. 2022), [18] (Kai et al. 2017), [19] (Yoon, Oh, and Park 2005), [20] (Li et al. 2014), [21] (Tsubouchi et al. 2013), [22] (Sanz‐Sáez et al. 2023), [23] (Hong et al. 2015), [24] (Angelova et al. 2021), [25] (Jin et al. 2015), [26] (Ivars‐Martinez et al. 2008), [27] (Jroundi et al. 2020), [28] (Chiu et al. 2007),[29] (Chan et al. 1978), [30] (Shao et al. 2010), [31] (Sánchez‐Porro et al. 2010), [32] (Kaye et al. 2011), [33] (Cai et al. 2019), [34] (Cui et al. 2008), [35] (Romanenko et al. 2008), [36] (Radisic et al. 2021),[37] (Ma et al. 2021), [38] (Yang 2018), [39] (Yoon, Lee, et al. 2005), [40] (Staloch et al. 2022), [41] (Heuchert et al. n.d.), [42] (Yumoto et al. 2003), [43] (Lee et al. 2008), [44] (Abdel‐Mageed et al. 2020), [45] (Ettoumi et al. 2013), [46] (Ruger and Koploy 1980), [47] (Cai et al. 2015), [48] (Bryan and Bryan 1959), [49] (Zhu et al. 2022), [50] (Alemán‐Vega et al. 2020), [51] (Ganesh Kumar et al. 2021), [52] (Fu et al. 2021), [53] (Ponomareva et al. 2022), [54] (Joshi et al. 2022), [55] (Gutiérrez‐Almada et al. 2020), [56] (Nedashkovskaya et al. 2010), [57] (Nedashkovskaya et al. 2005), [58] (Wei et al. 2018).

### 2.2 Bacterial Communities Structure

**FIGURE 4:**
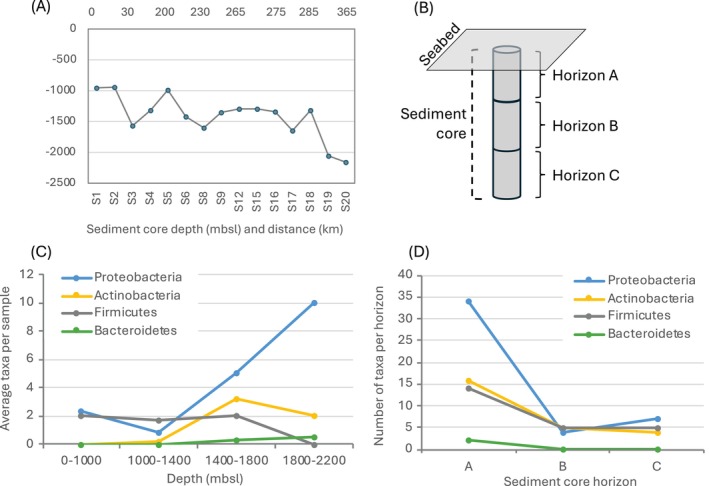
Occurrence of bacterial taxa at different depths and sediment horizons. (A) Sediment cores depth and distance from the first sample. (B) Graphical explanation of sediment cores division in horizons: Horizon A = seabed to −10 cm; Horizon B = −10 to −20 cm; Horizon C =−20 to −30 cm. (C) Average number of taxa belonging to phyla Proteobacteria, Actinomycetes, Firmicutes, and Bacteroidetes isolated from each sediment sample from four depth ranges. (D) Number of taxa belonging to four phyla isolated from different sediment horizons: A = 0 cm; B = −10 cm; C = −20 cm below sea bottom.

Sediment cores were sampled at depths ranging 933 to 2150 mbsl (Figure 4A), and bacteria were isolated from three different sections of each sediment core: horizons A, B, and C (Figure 4B). The number of bacterial taxa belonging to different phyla in each sediment core showed variability associated with increasing marine depth (Figure 4C) and sediment horizon depth (Figure 4D). Three phyla showed a higher number of different isolates in cores below 1000 mbsl: Pseudomonadota, Bacteroidetes, and Actinobacteria, with the first two showing the highest number at the deepest sampling sites (1800–2200 mbsl). Firmicutes showed a stable number of taxa from 900 to 1800 mbsl and no isolates at the deepest sites. All five phyla showed a considerably higher colonisation of surface horizons of each sediment (horizon A) compared to the deeper horizons. Actinobacteria and Firmicutes showed three times fewer isolates in horizons B and C compared to A, while Bacteroidetes was only isolated from the surface horizon A.

The diversity of bacteria isolated from different cores was evaluated using multivariate statistical analysis. PERmutational Multivariate ANOVA (PERMANOVA) was used to test the difference in bacterial communities between samples grouped with several parameters. A significantly different bacterial community was observed on different sediment core horizons (A, B or C; *p* = 0.046), while no significant difference was observed in sediments grouped by geographic location (*p* = 0.332), depth of sampling (*p* = 0.157), temperature at the site of sampling (*p* = 0.739) or sediment composition (*p* = 0.491).

The SIMilarity PERcentage (SIMPER) analysis was used to identify bacterial taxa mainly contributing to the differentiation of communities associated with deep‐sea sediments. Major contributors to dissimilarity between bacterial communities isolated from the three different horizons were always *Bacillus licheniformis* and *B. firmus* , respectively contributing on average 15% and 9.6%. The same test was also used to evaluate the species contribution to dissimilarity between grouped samples, with results reported in Table 3.

**TABLE 3:** Species contribution to bacterial community dissimilarity calculated by SIMPER.

Sample	Comparison	*Taxa*	Contribution %
Sediment type	Sandy mud/sand	*Microbacterium oxydans*	40.2
		*Bacillus licheniformis*	8.8
	Muddy sand/sand	*Bacillus licheniformis*	43.1
		*Leucobacter* sp.	8.6
	Muddy sand/sandy mud	*Bacillus licheniformis*	12.5
		*Halomonas meridiana*	7.6
Sediment horizons	Horizons: A/B	*Bacillus licheniformis*	11.9
		*Bacillus firmus*	7.4
	Horizons: B/C	*Bacillus licheniformis*	18.8
		*Bacillus firmus*	13.7
	Horizons: A/C	*Bacillus licheniformis*	14.4
		*Bacillus firmus*	7.7
Depth sections	900–1300/1300–1700	*Bacillus licheniformis*	11.4
		*Microbacterium oxydans*	8.8
	1300–1700/1700–2200	*Sulfitobacter pontiacus*	9.2
		*Pseudomonas* sp.*2*	7.2
	900–1300/1700–2200	*Sulfitobacter pontiacus*	9.6
		*Pseudomonas* sp.*2*	7.5

*Note:* SIMPER analyses were performed with PAST software.

Non‐metric Multi‐Dimensional Scaling (NMDS) was performed using the Bray–Curtis dissimilarity index (data not shown) and environmental variables such as depth, temperature, salinity, pH, and sediment composition; however, the model failed to generate a reliable dispersion of the data (stress values above 0.3).

### 2.3 Isolation Performance

Bacteria were isolated on media with different compositions and at two different temperatures to improve the chances of isolation. We implemented non‐selective nutrient‐rich media as well as nutrient‐poor and selective media. The vast majority of bacterial morphotypes isolated in this study grew on the nutrient‐rich medium ISP2 (75%), while 22% grew on nutrient‐poor media such as M4 (10%), BAPS (8%), and M2 (4%). Only five isolates (3% of bacterial isolates) were cultivated on the selective medium Gause's n°1 containing antimicrobial agents. Considering the temperature of isolation, 89% of bacterial isolates grew at 24°C, while only 11% grew at 4°C, and 8 taxa (14%) were isolated from both temperatures.

### 2.4 Occurrence of Biosynthetic Gene Clusters by Genetic Screening

PCR screening was conducted on bacterial isolates to evaluate the presence of genetic domains involved in the synthesis of bioactive metabolites. We targeted the amplification of ketosynthase (KS) domains belonging to polyketide synthase type II systems (PKS II‐KS), KS and methylmalonyl transferase (MMT) domains from PKS I systems (PKS I‐KS/MMT), and adenylation (A) domains in non‐ribosomal peptide synthases (NRPS‐A). Out of 139 bacterial isolates screened, 48 (35%) harboured at least one biosynthetic domain (PKS II‐KS, PKS I‐KS/MMT, or NRPS‐A); 19 isolates (14%) harboured 2 domains, and 4 isolates (3%) harboured 1 domain. No domain was detected in 68 genomes (49%).

**FIGURE 5:**
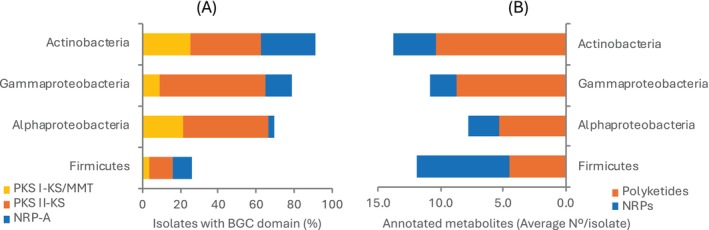
Biosynthetic profile of bacterial phyla isolated from deep‐sea sediments. (A) Genomic occurrence of biosynthetic gene cluster domains such as polyketide synthase type I ketosynthase and methylmalonyl transferase domains (PKS I‐KS/MMT), polyketide synthase type II ketosynthase domain (PKS II‐KS) and non‐ribosomal peptide synthase adenylation domain (NRPS‐A). Data indicate the percentage proportion of isolates carrying each biosynthetic domain. (B) Occurrence of polyketides and non‐ribosomal peptides (NRPs). Data indicate the average number of different molecules detected for each phylum by mass‐spectrometry, dereplication, and annotation.

All three genetic domains were detected in Actinobacteria, Alphaproteobacteria, Gammaproteobacteria, and Firmicutes (Figure 5A). Actinobacteria was the phylum with the highest proportion of isolates with detected biosynthetic domains (91%), followed by the classes Gammaproteobacteria (79%) and Alphaproteobacteria (70%), and phylum Firmicutes (26%). Among these phyla, PKS II‐KS was the most abundant, followed by PKS I‐KS/MMT and NRPS‐A.

All families in the phylum Actinobacteria showed isolates with biosynthetic domains: *Streptomycetaceae* (100%, 1 isolate), *Dietziaceae* (100%, 2 isolates), *Nocardiaceae* (75%, 4 isolates), *Microbacteriaceae* (71%, 17 isolates), and *Micrococcaceae* (38%, 8 isolates). In the class Gammaproteobacteria, the highest proportion was found in *Pseudomonadaceae* (83%, 12 isolates), *Halomonadaceae* (78%, 18 isolates), and *Moraxellaceae* (17%, 6 isolates), while the family *Alteromonadaceae* was not positive for any domain. In the class Alphaproteobacteria, all families showed isolates carrying biosynthetic genes, and the highest number of detected domains was found in *Hyphomonadaceae* (100%, 1 isolate), followed by *Rhodobacteraceae* (71%, 7 isolates), *Erythrobacteraceae* (50%, 22 isolates), and *Aurantimonadaceae* (50%, 2 isolates). All isolates (31) in the phylum Firmicutes belonged to the family *Bacillaceae*, and 17% carried at least one biosynthetic domain.

### 2.5 Deep‐Sea Bacteria Metabolomics

We implemented a metabolomic approach on 36 selected isolates to assess the biosynthesis of bioactive compounds. Tandem mass spectrometry data were analysed using the GNPS database for molecular networking and dereplication with the tool Dereplicator+. The obtained dataset of annotated molecules was further analysed with the ontology tool Classyfire to add molecular classification and then manually. All ethyl acetate extracts contained metabolites that dereplicated in the database. We found 9733 nodes assembled in GNPS, of which 1567 were known molecules annotated by Dereplicator+. Most of the known compounds (61.6%) belonged to lipid and lipid‐like molecules, of which almost half were represented by terpenoids. The second most abundant class of compounds was organic acids and derivatives (11.6%), of which over half were peptides. The superclass of phenylpropanoids and polyketides was the third most abundant (10.6%), but manual annotation was needed to include polyketides classified differently. Aromatic compounds such as alkaloids, benzenoids, lignans, and heterocyclic compounds represented 9.7% of the chemodiversity. The remaining 6.1% was formed by organic compounds containing nitrogen or oxygen as well as small hydrocarbons. Among bacterial phyla, major producers of polyketides were Actinobacteria, which synthesised over 10 known molecules per strain on average, followed by Gammaproteobacteria (nine molecules), Alphaproteobacteria (five molecules) and Firmicutes (less than five molecules). Conversely, major producers of peptides were Firmicutes, with over seven known compounds synthesised per strain, followed by Actinobacteria (over three molecules), Alphaproteobacteria (over two molecules), and Gammaproteobacteria (two molecules, Figure 5B).

**FIGURE 6:**
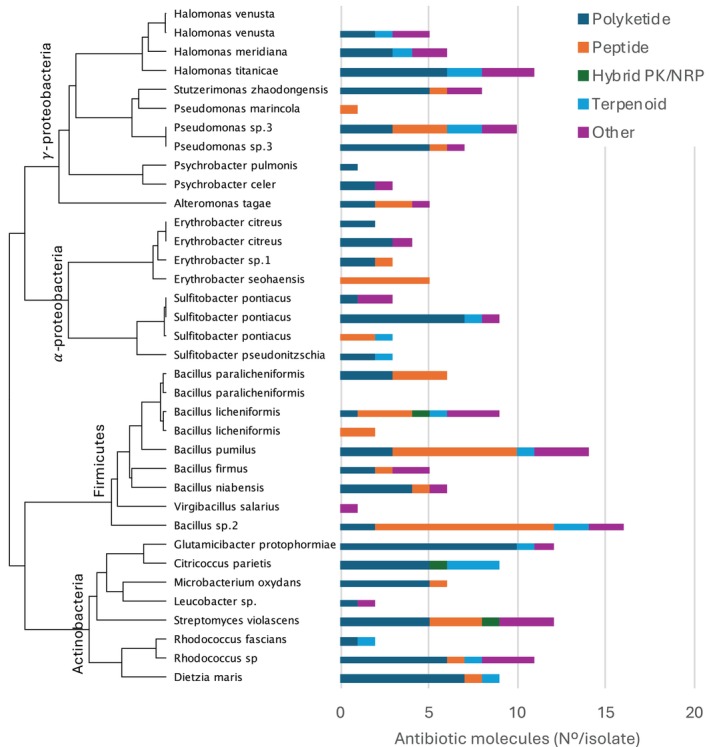
Antibiotic molecules synthesised by a selection of deep‐sea bacteria. Sediment isolates were axenically cultivated for 14 days in their preferred growing medium prepared in seawater. Secondary metabolites were then extracted with ethyl acetate and analysed with tandem mass‐spectrometry. Molecule dereplication was performed with Dereplicator+ in GNPS, while annotation was done with Classyfire. The phylogenetic tree on the left is composed of bacterial species studied for chemical synthesis, their phylogenetic correlation, and phylum (Gammaproteobacteria, Alphaproteobacteria, Firmicutes, and Actinobacteria). The bar‐chart on the right indicates the number of different antibiotics detected from each isolate, divided by molecular class (polyketide, peptide, hybrid PK/NRP, terpenoid, other).

The chemical dataset was manually annotated to assess the presence of known antibiotics or compounds with putative quorum sensing function such as gamma‐butyrolactones. About half of the annotated antibiotics (101 out of 211) have literature evidence of polyketide structure or biosynthesis by polyketide‐like pathways. The second most abundant antibiotics were peptides (49), which included 25 depsipeptides, 20 lipopeptides, and four polypeptides. Three antibiotic compounds were hybrid non‐ribosomal peptides/polyketides, while terpenoids provided 20 antibiotic molecules, belonging to terpene glycosides (9), polyterpenoids (7) and terpene lactones (4). A number of 38 not structurally annotated antibiotics were also detected. The highest amount of dereplicated antibiotic molecules was synthesised by Actinobacteria (63 compounds), followed by Firmicutes (59), Gammaproteobacteria (57) and Alphaproteobacteria (32). Several species belonging to each investigated phyla showed higher than average biosynthesis of antibiotics: *Streptomyces violascens* (12), *Glutamicibacter protophormiae* (12) and *Rhodococcus* sp. (11) in the phylum Actinobacteria; *Bacillus* sp.2 (16) and *B. pumilus* (14) in the phylum Firmicutes; one strain of *Sulfitobacter pontiacus* (9) in the class Alphaproteobacteria; *Halomonas titanicae* (11) and one strain of *Pseudomonas abyssi* (10) in the class Gammaproteobacteria (Figure 6).

**FIGURE 7:**
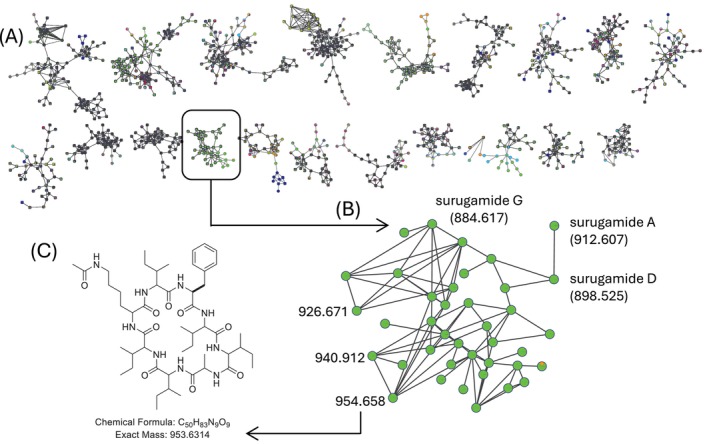
Microbial extracts metabolomic analysis by molecular networking. (A) Portion of the MS/MS molecular network of deep‐sea microbial extracts elaborated by GNPS. The circled cluster of 39 nodes was selected as it is composed of molecules produced by one single organism, *Streptomyces violascens* . (B) The selected cluster is composed of known surugamides that dereplicated in the database as well as non‐dereplicated compounds with structural similarity. Nodes with m/z values 926.671, 940.912 and 954.658 were investigated for molecular novelty, but only the molecule with mass 954.658 was biosynthesised in a concentration that allowed purification. (C) Structure elucidation of the purified molecule by two‐dimensional mass spectrometry indicated a compound with the name acyl‐surugamide A2.

In an attempt to probe for previously unreported compounds, we inspected the metabolites network generated by GNPS and searched for clusters of molecules potentially indicating the synthesis of new bioactive compounds. We selected a cluster of nodes in the network that was only present in one organism's extract, a sign of specialised metabolism. The cluster we selected was formed by 39 nodes produced by *Streptomyces violascens* and contained nodes annotated by GNPS as non‐ribosomal peptides surugamide A, surugamide D, and surugamide G. Among the nodes in this cluster with unreported structure, one with the mass 954.6443 was recently described (Maw et al. 2024) as acyl‐surugamide A2. This molecule was the only one in the surugamide cluster to be synthesised at a higher concentration and therefore the only molecule we were able to isolate, purify, and characterise (Figure 7).

## 3 Discussion

### 3.1 Cultivation of Bacteria From Deep North Atlantic Ocean Sediments

In this study, 15 seafloor sediment cores sampled at the edge of the Porcupine Bank in the Irish Atlantic Ocean were processed for bacterial isolation to provide evidence of distinctive species diversity and evaluate the biosynthesis of chemical signals such as antibiotics and quorum sensing compounds. Data generated in our survey confirm wide bacterial colonisation of deep‐sea sediments in the North Atlantic Ocean: all samples obtained from a geographical area spanning about 360 km in length at depths ranging from 933 to 2150 mbsl showed the presence of culturable bacteria. The number of strains isolated from each sediment core in our study (9.6 on average) was similar to that reported from the South Atlantic with an average of 9 isolates per sediment core (da Silva et al. 2013). A factor potentially biasing the number of bacterial isolates is the cryopreservation of our samples compared to the above‐mentioned study, which stored sediments at 4°C before isolation. Biotic factors such as the sediment organic carbon content might have also influenced higher microbial abundance in the South Atlantic compared to the North Atlantic Ocean. As previously demonstrated, total organic carbon content as well as the chemical composition or organic carbon source in deep‐sea sediments significantly influences the development of bacterial communities (Bienhold et al. 2016; Goffredi and Orphan 2010; Hoffmann et al. 2017).

The cultivation of deep‐sea bacteria was performed to provide optimal and variable growth conditions to lineages with different requirements, aimed at maximising the number of isolates obtained. The cultivation medium providing the highest number of isolates was ISP2, a nutrient‐rich nonselective medium, while most isolates (89%) were isolated from plates incubated at 24°C, a considerably higher temperature than the sampling site. Results from our study also showed nine taxa growing from nutrient‐poor or selective media incubated at 4°C, demonstrating that variation of cultivation conditions can lead to over 15% increase in taxa isolated from environmental samples.

### 3.2 Bacterial Community Composition

The bacterial diversity we report in this study shows similarities with previous worldwide reports indicating Pseudomonadota as the dominant lineage of deep ocean sediments (Bienhold et al. 2016; Cerqueira et al. 2015; da Silva et al. 2013; Reese et al. 2018; Trouche et al. 2021; Zinger et al. 2011). In our study, the majority of Pseudomonadota were affiliated with the Gamma‐lineage, followed by Alphaproteobacteria, and no isolates belonging to other lineages were detected. This contrasts with global patterns which showed Delta‐ and Beta‐proteobacteria to significantly contribute to sediment bacterial diversity (Bienhold et al. 2016; Zinger et al. 2011) but highlights a higher diversity compared to a previous study in the Atlantic Ocean only reporting the cultivation of Gammaproteobacteria (da Silva et al. 2013). Deltaproteobacteria, currently known as Myxobacteria, are characterised by a typical multicellular organisation allowing their movement in the direction of attractants and a predatory behaviour towards other microbes (Wrótniak‐Drzewiecka et al. 2016). Their predatory trophic preference requires isolation techniques involving bait bacteria (Shimkets et al. 2006) which likely impacted on their growth using media designed for saprotrophic species. Considering Betaproteobacteria, the majority of its members belong to mutualistic symbionts of animals, plants, and fungi (Degli Esposti et al. 2018), while a group of marine inhabitants called OM43 typically exhibits methylotrophic behaviour (Huggett et al. 2012); in both cases, lifestyles requiring specific conditions for cultivation.

Considerable contribution to the bacterial diversity in this study was provided by Actinobacteria and Firmicutes: these two phyla showed a number of isolates two to three times higher than previously reported global patterns (Bienhold et al. 2016; Zinger et al. 2011), but similar results to what has been reported by cultivation‐driven surveys in the Atlantic and Pacific Oceans (Blandón et al. 2022; da Silva et al. 2013; Romanenko et al. 2013). A smaller proportion of the overall community was represented by Bacteroidetes, which was not reported in previous cultivation studies from the Atlantic Ocean but showed a smaller rate in comparison with global patterns and a cultivation‐based study in the Pacific Ocean (Bienhold et al. 2016; Romanenko et al. 2013; Zinger et al. 2011).

The higher proportion of Actinobacteria and Firmicutes detected in our study in comparison with global patterns could be explained by the different methodologies used to study bacterial diversity, which were culture‐independent in the latter case (Bienhold et al. 2016; Zinger et al. 2011). In our and other culture‐driven studies (Blandón et al. 2022; da Silva et al. 2013; Romanenko et al. 2013), bacteria with less selective environmental requirements as well as with the ability to form spores are more likely to thrive in laboratory conditions compared to other members of the microbiota with selective needs. In support of this hypothesis is the evidence that Actinobacteria as well as Firmicutes can form spores, and are remarkable environmental saprophytes with the ability in the case of some Actinobacteria to decompose recalcitrant organic matter, allowing their distribution in many worldwide environments (Barka et al. 2016). Another possible explanation for the variable abundances of bacterial phyla between cultivation and metabarcoding‐based studies is the lower taxonomic resolution provided by previous culture‐independent methods used in global surveys (Bienhold et al. 2016; Zinger et al. 2011), which potentially led to underestimating the abundance of bacterial lineages with low microbial load and high biodiversity. From a methodological perspective, our samples cryopreservation before bacterial isolation might have introduced changes in the community structure leading to a biased recorded biodiversity. As previously observed, bacteria belonging to different phyla and classes variably respond to cryopreservation, with a general decline in diversity recorded after storage, which severely affects Bacteroidetes and is less impactful on Pseudomonadota (M. Park et al. 2021). However, a comprehensive study assessing the viability of marine bacteria after cryopreservation highlighted that between 45% and 72% of harvested aquatic communities survive freezing, if appropriate sample processing is performed (Rain‐Franco et al. 2021). The use of glycerol as cryoprotectant was shown to be effective in preserving viable bacteria from raw marine samples, leading to a successful isolation and cultivation of multiple cultures of SAR11 marine bacteria which are known to be difficult to cultivate (Monaghan et al. 2020).

Phylogenetic analysis of isolated bacteria using full‐length 16 s rDNA genes including all nine variable regions allowed clustering of most isolates with type strain sequences and their identification at the species level. Thirteen taxa were only assigned at the genus level as they showed high sequence similarity with multiple taxa in the database and clustered independently within the phylogenetic trees. Moreover, two isolates showing low sequence similarity in the database and independent segregation in the phylogenetic tree were assigned at the genus level and represent potential new species. Considering the environmental segregation of deep‐sea sediments, the vast extension of such an environment, and the limited number of cultivation studies, it is highly probable that the deep‐sea harbours new bacterial species.

### 3.3 Drivers of Bacterial Diversity in Deep‐Sea Sediments

Culturable bacteria diversity in our study increased at deeper sampled sites, with a higher number of phyla and taxa detected below 1000 mbsl. This contrasts with a previous report from the North Atlantic Ocean highlighting decreasing bacterial diversity with increasing depth, especially at the transition between 800 and 1200 mbsl (Trouche et al. 2021). However, other authors indicated geographical location as a stronger driver of bacterial diversity in deep‐sea sediments compared to depth (Zinger et al. 2011), while sediment organic carbon availability and composition were major drivers in other studies (Bienhold et al. 2016; Goffredi and Orphan 2010; Hoffmann et al. 2017). Sediments investigated in this research were collected on a slope connecting the Porcupine Bank with the abyssal plain, an area characterised by irregular geomorphology and numerous canyons influencing variable deposition of organic carbon, which likely influences the formation of different ecological niches driving local biodiversity. Microbial species detected below 1000 mbsl included several bacteria that were first described from the deep‐sea environment, including several members of the genus *Psychrobacter* such as *P. celer, P. nivimaris*, and P. *okhotskensis*, a genus generally known for its adaptation to extreme habitats characterised by cold temperature (Rodrigues et al. 2009), such as those recorded in the deep sea.

Major contributors to bacterial diversity were tested with SIMPER, which highlighted *Bacillus* species significantly influencing diversity on different sediment horizons, cores with different composition, and at different depths. *Sulfitobacter pontiacus* characterised the diversity of cores at the deepest sites, while *Microbacterium oxidans* showed a strong influence in characterising sand cores. Members of the family *Bacillaceae* are endospore‐forming bacteria with remarkable resistance to environmental stress that have key roles in soil ecology (Siala et al. 1974; Toljander et al. 2006) and were previously highlighted as a dominant lineage in shallow water marine sediments (Zinger et al. 2011). Due to their role and wide soil distribution, their benthic abundance and resistance to stress, *Bacillus* isolates could be active soil bacteria that are washed into the marine environment and survive inactive in the deep sea. However, the presence of isolates potentially belonging to new species might indicate non‐indigenous deep‐sea taxa that are active members of the benthic environment. *Sulfitobacter* is an ecologically relevant and widespread marine genus, often isolated from marine invertebrates (Ivanova et al. 2004; Kumari et al. 2016) as well as associated with microalgae (Hong et al. 2015), sediments (Park et al. 2007) or floating at abyssal depth (Sanz‐Sáez et al. 2023). *Sulfitobacter pontiacus* is a widespread species with an ecological role in the sulfur cycle (Moran et al. 2003) that was first isolated from the Black Sea (Sorokin 1995) and later detected abundantly in the bathypelagic zone of the world's oceans using both culture independent and cultivation strategies (Sanz‐Sáez et al. 2023). In this study, we prove the genetic and metabolic ability of *Sulfitobacter* species to biosynthesise an array of polyketides with previously demonstrated antibiotic function, which might provide them with the necessary competitive advantage to colonise a wide range of marine habitats.

We ultimately evaluated the influence of environmental factors such as geographical location, sediment grain size, depth or horizon in shaping the bacterial community, but none of them significantly influenced community structure. Bacteria isolated from the processed samples were mostly present on one single sediment core, negatively influencing the multivariate statistics and causing a very high evenness associated with the datasets (Forcino et al. 2015). Further studies should implement additional strategies for bacteria cultivation such as hydrostatic pressure to mimic their natural environment, while characterisation of the organic carbon content and composition of each sample would provide valuable information on the biotic factors potentially influencing biodiversity.

### 3.4 Bioactive Compound Synthesis by Deep Sea Bacteria

We investigated the ability of sediment‐inhabiting bacteria to synthesise bioactive secondary metabolites. Even though we detected BGCs in only half of our isolates' genomes, the vast majority of bacteria analysed with metabolomics were found to synthesise multiple known secondary metabolites. Actinobacteria secreted up to 12 different known bioactive compounds per strain in axenic culture, all known for their antibiotic and quorum sensing activities. An isolate of *Streptomyces violascens* also synthesised a unique range of compounds not produced by any other bacteria in this study, including known Surugamide compounds and new analogues. A previously unknown and now described Surugamide analogue (Maw et al. 2024) that we isolated was produced in considerable amount (~0.1 mg/L), indicating that the organism engages a substantial amount of energy in the biosynthesis of a range of molecules with the hypothetical primary aim to outcompete other organisms. Benthic deep‐sea is an extreme environment with low nutrients availability, where the secretion of antagonistic molecules provides a considerable strategic advantage to thrive over other residing microbes. Such behaviour, called interference competition, was already observed in marine sediment actinomycetes as a species‐specific physiological strategy (Patin et al. 2017) as well as in hypersaline microbial mats where it influenced the bacterial community structure (Long et al. 2013). Actinobacteria are known for their remarkable production of antibiotics (Millán‐Aguiñaga et al. 2019), with the genus *Streptomyces* that is estimated to biosynthesise up to 100,000 antimicrobial compounds (Salwan and Sharma 2020; Watve et al. 2001). In this study, we confirm the outstanding ability of such genus to secrete known and new bioactive metabolites, while further physiological studies are required to underpin the ecological role of unique compounds. Species belonging to Pseudomonadota lineages showed a lower number of known antibiotics and quorum sensing molecules detected by chemical screening per isolate, and no isolate showed to synthesise unique sets of compounds generating independent clusters. This phylum has previously demonstrated to synthesise ecologically relevant polyketides (Bender et al. 1999), and to harbour biosynthetic gene clusters likely producing an array of bioactive compounds (Buijs et al. 2019). However, Pseudomonadota has attracted considerably less interest than Actinobacteria for natural products discovery, possibly generating a lack of reference molecules and associated functions in public databases. Considering that this phylum is widespread in deep‐sea sediment microbial communities, it is necessary to improve knowledge on their natural products biosynthesis. Chemical extraction and characterisation of unknown molecules will provide data to feed reference databases and support annotation, facilitating future chemical ecology studies.

Bacteria belonging to the Firmicutes lineage showed remarkable biosynthesis of antibiotic peptides rather than polyketides. Among the investigated taxa, one unidentified isolate here reported as *Bacillus* sp.2 and one isolate of *Bacillus pumilus* , secreted an over two‐fold variety of bioactive peptides compared to other tested strains. Both taxa produced a range of surfactin analogues, while *Bacillus* sp.2 showed specialisation in the biosynthesis of iturin, with five known secreted analogues of these pore‐forming lipopeptides with antibiotic activity against bacteria and fungi (Besson et al. 1978; Han et al. 2024). Bioactive lipopeptides synthesised by *Bacillus* and other genera are considered prominent components of the core specialised metabolome (Balleux et al. 2025), in which bacteria invest considerable amount of resources for their production even in nutrient‐limited conditions (Andrić et al. 2023; Hoff et al. 2021). Their function is well characterised in soil environment, with ecological roles spanning signalling for biofilm formation, competition, and symbiosis establishment (Balleux et al. 2025). Several previously unknown bioactive lipopeptides were discovered from deep‐sea bacteria in recent years (Ganesh Kumar et al. 2021; Zhou et al. 2019; Zhou et al. 2020), but their role under extreme environmental conditions was not investigated. Drawing parallels from the terrestrial environment, such specialised molecules might be synthesised in the deep‐sea to provide strategic advantage through interference competition or to improve colony fitness by biofilm formation (Deschaine et al. 2018).

Overall, a substantial number of deep‐sea isolates demonstrated to possess genomic features enabling the biosynthesis of known bioactive compounds and to invest considerable metabolic activity in the biosynthesis of antibiotics or quorum sensing signals. The higher number of annotated compounds per molecular class could be due to over‐annotation of data from mass spectrometry by the dereplication tools, which potentially assigned intermediate products of biosynthesis or analogues. However, considering the demonstrated role of bacterial bioactive compounds in communicating with other organisms in aquatic and soil habitats (Biarnes‐Carrera et al. 2015; Iwasa et al. 1998; Kim et al. 2023; Patin et al. 2017), it is reasonable to predict that some bioactive molecules synthesised by each deep‐sea bacterial strain was secreted with the same purpose and contributed to shaping deep‐sea communities. Additional experiments are required to prove the biosynthesis of such compounds in the natural habitat and underpin their ecological role at the community level.

## 4 Conclusions

Our study highlights sediments in the deep‐sea of the North Atlantic Ocean populated by bacterial communities with species already known as deep‐sea dwellers, along with taxa previously unknown for their adaptation to this extreme environment. We applied a culturomics approach, which allowed for detailed taxonomic assignments as well as investigations into the physiology and genomics of deep‐sea bacteria. We recorded a culturable biodiversity dominated by species belonging to the phyla Pseudomonadota, Actinobacteria, and Firmicutes; while the most diverse families were the *Bacillaceae*, *Alteromonadaceae, Pseudomonadaceae*, *Erythrobacteriaceae*, and *Microbacteriaceae*. Most strains were isolated on nutrient‐rich non‐selective media (ISP2), but diversification of culture conditions was necessary to isolate strains with different growth requirements. According to our study, bacteria were unevenly distributed across the sampling locations, with only species belonging to *Bacillaceae* present on more than half of the sediment samples. This might be due to very different environmental conditions associated with sediments at different sampling sites. However, technical limitations of the culture‐dependent method as well as sample variability induced by freeze‐thawing likely biased our representation of the samples' biodiversity.

Bacteria isolated from deep‐sea sediments had the ability to synthesise a wide range of bioactive compounds, including known antibiotics and quorum sensing molecules. Almost all species belonging to *Actinobacteria* had the genomic signature for polyketides and/or non‐ribosomal peptides biosynthesis, whose production was confirmed with metabolomic investigation. While Actinobacteria and Pseudomonadota had a remarkable competence in the biosynthesis of polyketides rather than non‐ribosomal peptides, Firmicutes showed an inverted trend with a higher production of non‐ribosomal peptides. Some species showed remarkable biosynthetic power compared to other strains in the same taxonomic groups, highlighting potentially species‐specific strategies for interference‐competition. However, sampling cryopreservation might have affected the biosynthetic capabilities of certain species, skewing our understanding of strain‐associated secondary metabolites biosynthesis. A thorough understanding of the chemical interplay within microbial communities has yet to be reached, and additional in vitro experiments are required to address the specific role of each bioactive molecule. However, the isolation of native deep‐sea bacteria is crucial for studying the physiology of organisms from extreme environments and providing microbial strains for biotechnology development.

## 5 Materials and Methods

### 5.1 Deep‐Sea Sediment Sampling and Processing

Deep‐sea sediments were collected during cruise ce18012 in the North Atlantic Ocean using the Remotely Operated Vehicle (ROV) Holland I onboard the Research Vessel (R/V) Celtic Explorer (Figure 1, Table 1). A conductivity‐temperature‐depth (CTD) profiler (Sea‐Bird Electronics) mounted on the ROV was used to record environmental parameters. Fifteen sediment push cores were obtained from different sites on the Porcupine Bank northern edge (sites 1–20 on Figure 1), at depths ranging between 933 and 2150 m below sea level (mbsl). Sediment cores were aseptically processed on board, divided into horizons of 10 cm length: horizon A (seabed/−10 cm), horizon B (−10 cm/−20 cm) and horizon C (−20 cm/−30 cm). Each horizon sample was added to diluted sterile glycerol in equal volumes of sediment solution to a final concentration of 15% for cryopreservation and stored in 50 mL tubes at −80°C. Sediment composition was evaluated by particle size analysis measuring the proportion of different components with Laser Particle Sizing in a Malvern Mastersizer 2000 and classified as sand, muddy sand, or sandy mud. For detailed procedure see previous work (Marchese et al. 2021).

### 5.2 Isolation of Bacteria From Sediments

Bacteria were isolated from three separate horizons (A, B and C) of each sediment core. For microbial growth, approximately 1 g of thawed sediment from each section was diluted in 9 mL sterile Dulbecco's Phosphate Buffered Saline solution, vortexed and incubated on a tumble‐shaker for 1 h. In total, 45 samples derived from three horizons per sediment were inoculated on four agar plates per medium (ISP2, Gause's n1, BAPS, M2, M4; see Table S1 for media formulation) by spread plating 0.1 mL of sample and incubated at 5°C (two plates) and at 24°C (two plates). The first week after inoculation, plates were inspected daily for microbial growth, then once weekly up to 8 weeks. Following morphological inspection of bacterial colonies growing on isolation plates, one isolate per colony morphotype was isolated in pure culture and stored at −80°C in glycerol solution.

### 5.3 Bacteria Identification

Genomic DNA was extracted from axenic bacterial isolates using the DNeasy Blood and Tissue Kit (Qiagen). The full‐length 16S rDNA gene was amplified by PCR using the forward primer FC27 (5′‐AGAGTTTGATCCTGGCTCAG‐ 3′) and reverse primer RC1492 (5′‐TACGGCTACCTTGTTACGACTT‐3′). PCR reactions were cleaned using the QIAquick PCR Purification Kit (Qiagen) and both strands of purified amplicons were sequenced using Sanger technology. Sequences were then aligned in the GenBank (nBlast; mismatch 1/−2; gap costs linear) database, and a list of possible species matches was compiled. Taxonomic identity was further investigated and confirmed by phylogenetic analysis: taxa were grouped by family, and datasets were populated with type strains' sequences of possible species matches. Sequence alignment was performed using MUSCLE on Mega X (Kumar et al. 2018; Stecher et al. 2020), using default conditions for gap openings and gap extension penalties, with manual adjustments. Phylogenetic analyses were performed in Mega X using the Maximum Likelihood method with the General Time Reversible Model or Jukes Cantor model and 1000 bootstrap iterations.

### 5.4 Diversity Analysis

Bacterial diversity within samples was measured with the Shannon‐Weaver index (H′), Gini‐Simpson index (1‐Lambda) and Pielou's evenness (J'). Differences in diversity within sampling sites were calculated using Permutational Multivariate Analysis of Variance (PERMANOVA; pseudo‐F index; *p* < 0.05) with samples grouped by geographic location, depth, temperature, and sediments composition. The Similarity Percentage test (SIMPER) was used to quantify the contribution of single species to the diversity between each set of samples. Statistical analyses were performed using PAST 4 for multivariate ecological studies (Hammer et al. 2001).

### 5.5 Bacteria Biosynthetic Domains Screening

Following a previously described methodology (Ayuso et al. 2005; Ayuso‐Sacido and Genilloud 2005), bacterial genomes were screened for the presence of Biosynthetic Gene Cluster domains. Degenerate primers K1F and M6R (5′‐TSAAGTCSAACATCGGBCA‐3′, 5′‐CGCAGGTTSCSGTACCAGTA‐3′) were used to target PKS‐I keto synthase and methyl malonyl transferase domains; A3F and A7R (5′‐GCSTACSYSATSTACACSTCSGG‐3′, 5′‐SASGTCVCCSGTSCGGTAS‐3′) to target Non‐Ribosomal Peptide adenylation domain; KSα and KSβ (5′‐TSGRCTACRTCAACGGSCACGG‐3′, 5′‐TACSAGTCSWTCGCCTGGTTC‐3′), to target conserved sequences in PKS‐II KSα and KSβ domains. PCR products were analysed by electrophoresis in 10% acrylamide gels and positive amplification of targets was assigned if bands of the appropriate size were detected: 1200–1400 bp for K1F and M6R primers, 600–700 bp for A3F and A7R primers, and 600–2100 bp for KSα and KSβ primers.

### 5.6 Bacteria Metabolites' Extraction and Metabolomic Analysis

Selected bacteria were cultivated in ISP2 or CSB liquid medium, in agitation at 24°C for 14 days. Visual inspection was performed to evaluate possible fungal contamination, and each culture was inoculated on solid medium to confirm original morphology. Metabolite extraction was performed by addition of a 1:1 volume of ethyl acetate to the fermented liquid medium, then vigorously shaken and separated in a separating funnel. The solvent extraction was performed three times with equal volumes of ethyl acetate, and solvent was removed by vacuum evaporation. Crude extracts were then cleaned with a C18 Sep Pak column (Waters): extracts were dissolved in 95% MeOH/5% H_2_O, loaded on the C18 column, washed three times with 5% MeOH/95% H_2_O, eluted with a solution of 95% MeOH/5% H_2_O, and dried in a nitrogen stream.

Bacterial extracts were dissolved in MeOH and analysed with Liquid Chromatography/quadrupole Time‐of‐Flight mass spectrometry (LC/qToF MS/MS) to gain molecular ion and fragmentation information about metabolites in each extract.

A dataset containing metabolomic profiles of all extracts was submitted for analysis to GNPS (Wang et al. 2016) and a molecular network was created in Cytoscape (Shannon et al. 2003). The dereplication tool Dereplicator+ (Mohimani et al. 2018) in GNPS was used to detect known metabolites in the dataset, while the annotation tool ClassyFire (Djoumbou Feunang et al. 2016) was used to provide classification to molecular features found in the mass spec data.

## Author Contributions

**Pietro Marchese:** conceptualization, investigation, writing – original draft, writing – review and editing, visualization, formal analysis, data curation, methodology, software, validation, funding acquisition, project administration. **Joe Bracegirdle:** investigation, methodology, writing – review and editing, software, formal analysis, conceptualization, validation. **Ryan Young:** resources, methodology, project administration. **Emanuele Ferrari:** writing – review and editing, validation. **Laura Garzoli:** writing – review and editing, validation. **J. Mary Murphy:** funding acquisition, supervision, writing – review and editing. **Maria Tuohy:** resources, funding acquisition, project administration. **A. Louise Allcock:** resources, writing – review and editing, project administration. **Bill J. Baker:** supervision, resources, project administration, writing – review and editing, funding acquisition.

## Conflicts of Interest

The authors declare no conflicts of interest.

## Supporting information


**Table S1.** Solid media formulation for the isolation of bacteria from deep‐sea sediments.
**Table S2.** List of bacterial isolates with Blast match and GenBank accession numbers.
**Figure S1.** Phylogenetic affiliation of isolates from the family Nocardiaceae.
**Figure S2.** Phylogenetic affiliation of isolates from the family Microbacteriaceae.
**Figure S3.** Phylogenetic affiliation of isolates from the family Micrococcaceae.
**Figure S4.** Phylogenetic affiliation of isolates from the family Erythrobacteraceae.
**Figure S5.** Phylogenetic affiliation of isolates from the family Hyphomonadaceae.
**Figure S6.** Phylogenetic affiliation of isolates from the family Aurantimonadaceae.
**Figure S7.** Phylogenetic affiliation of isolates from the family Rhodobacteraceae.
**Figure S8.** Phylogenetic affiliation of isolates from the family Halomonadaceae.
**Figure S9.** Phylogenetic affiliation of isolates from the family Alteromonadaceae.
**Figure S10.** Phylogenetic affiliation of isolates from the family Pseudomonadaceae.
**Figure S11.** Phylogenetic affiliation of isolates from the family Moraxellaceae.
**Figure S12.** Phylogenetic affiliation of isolates from the family Bacillaceae.
**Figure S13.** Phylogenetic affiliation of isolates from the family Flavobacteriaceae.

## Data Availability

The data that supports the findings of this study are available in the supplementary material of this article.
