# Supplementary material for: Bacterial Diversity in Deep‐Sea Sediments of the North Atlantic Ocean and Their Biosynthesis of Secondary Metabolites
Source: Environ Microbiol Rep. 2025 Jul 9;17(4):e70092. doi: 10.1111/1758-2229.70092 (PMC12239160; doi:10.1111/1758-2229.70092)
Supplement: Supplementary file 1 — Table S1. Solid media formulation for the isolation of bacteria from deep‐sea sediments. Table S2. List of bacterial isolates with Blast match and GenBank accession numbers. Figure S1. Phylogenetic affiliation of isolates from the family Nocardiaceae. Figure S2. Phylogenetic affiliation of isolates from the family Microbacteriaceae. Figure S3. Phylogenetic affiliation of isolates from the family Micrococcaceae. Figure S4. Phylogenetic affiliation of isolates from the family Erythrobacteraceae. Figure S5. Phylogenetic affiliation of isolates from the family Hyphomonadaceae. Figure S6. Phylogenetic affiliation of isolates from the family Aurantimonadaceae. Figure S7. Phylogenetic affiliation of isolates from the family Rhodobacteraceae. Figure S8. Phylogenetic affiliation of isolates from the family Halomonadaceae. Figure S9. Phylogenetic affiliation of isolates from the family Alteromonadaceae. Figure S10. Phylogenetic affiliation of isolates from the family Pseudomonadaceae. Figure S11. Phylogenetic affiliation of isolates from the family Moraxellaceae. Figure S12. Phylogenetic affiliation of isolates from the family Bacillaceae. Figure S13. Phylogenetic affiliation of isolates from the family Flavobacteriaceae. [file EMI4-17-e70092-s001.docx]

Supplementary information

Exploring Bacterial Diversity in Deep-Sea Sediments of the North Atlantic Ocean and Their Biosynthesis of Secondary Metabolites

Pietro Marchese^1,2,4^, Joe Bracegirdle^2^, Ryan Young^2^, Emanuele Ferrari^1^, Laura Garzoli^1^, J Mary Murphy^3^, Maria Tuohy^4^, A Louise Allcock^5^, Bill J Baker^2^

^1^Molecular Ecology Group, Water Research Institute, Italian National Research Council, Corso Tonolli 50, Verbania, Italy

^2^Department of Chemistry, University of South Florida, 4202 E. Fowler Avenue, CHE205, Tampa, FL 33620, USA

^3^Regenerative Medicine Institute, School of Medicine, University of Galway, Galway, H91TK33, Ireland.

^4^Molecular Glycobiotechnology, School of Natural Sciences, University of Galway, Galway, H91TK33, Ireland.

^5^Martin Ryan Institute, School of Natural Sciences, University of Galway, Galway, H91TK33, Ireland.

Table S1: Solid media formulation for the isolation of bacteria from deep-sea sediments

Table S2: List of bacterial isolates with Blast match and GenBank accession numbers

Figure S1: Phylogenetic affiliation of isolates from the family Nocardiaceae.

Figure S2: Phylogenetic affiliation of isolates from the family Microbacteriaceae.

Figure S3: Phylogenetic affiliation of isolates from the family Micrococcaceae.

Figure S4: Phylogenetic affiliation of isolates from the family Erythrobacteraceae.

Figure S5: Phylogenetic affiliation of isolates from the family Hyphomonadaceae.

Figure S6: Phylogenetic affiliation of isolates from the family Aurantimonadaceae.

Figure S7: Phylogenetic affiliation of isolates from the family Rhodobacteraceae.

Figure S8: Phylogenetic affiliation of isolates from the family Halomonadaceae.

Figure S9: Phylogenetic affiliation of isolates from the family Alteromonadaceae.

Figure S10: Phylogenetic affiliation of isolates from the family Pseudomonadaceae.

Figure S11: Phylogenetic affiliation of isolates from the family Moraxellaceae.

Figure S12: Phylogenetic affiliation of isolates from the family Bacillaceae.

Figure S13: Phylogenetic affiliation of isolates from the family Flavobacteriaceae.

*Phylogenetic affiliation of deep-sea isolates*

Taxonomic affiliation of bacteria isolated from the deep-sea was investigated by phylogenesis using the full-length 16s rRNA. Phylogenetic trees were constructed using sequences belonging to deep-sea isolates divided by phylum and family, along with sequences of type strains (t.s.) of species with >99% overlap and >97% similarity in the NCBI nucleotide Blast database. Sequences alignment and tree construction was performed in MEGA X.

Phylum Actinobacteria

**Figure S.1: Phylogenetic affiliation of isolates from the family Nocardiaceae.**

Maximum Likelihood phylogram of Nocardiaceae based on 16s rDNA. Three isolates belonging to the family Nocardiaceae grouped with *Rhodococcus fascians* t.s. DSM 20669, while one isolate (SFI-C161) segregated independently in a closely related clade and was ultimately assigned as *Rhodococcus* sp. One isolate belonging to the *Nocardioides* genus (SFI-C107) grouped together with *N. dokdonensis*, in a branch with remarkable genetic distance and therefore assigned as *Nocardioides* sp. Two isolates of *Dietzia* were confirmed as *D. maris* by comparison with the t.s. DSM 43672. One isolate of *Streptomyces* was confirmed as *S. violascens* in accordance with NCBI database match. *Bacillus aerius*. Phylogenetic inference was performed using MEGA X with 1000 bootstraps and the General Time Reversable method.

**Figure S.2: Phylogenetic affiliation of isolates from the family Microbacteriaceae.**

Maximum Likelihood phylogram of Microbacteriaceae based on 16s rDNA. Five isolates showing high database sequence similarity with *Microbacterium oxydans* grouped together with the t.s. DSM 20578; while other six isolates with high database sequence similarity (SFI-C25, SFI-C75, SFI-A10, SFI-A5, SFI-C152, SFI-C94), grouped together in an independent and closely related clade and were assigned as *Microbacterium* sp. Two isolates grouped together with *M. profundi* and were taxonomically confirmed. One isolate with high database sequence similarity with *Leucobacter komagatae* (SFI-N11) was assigned as *Leucobacter* sp. as it did not group with *L. komagatae* t.ss. or other members of the genus. Three isolates were confirmed as *Salinibacterium amurskyense* by comparison with the t.s. KMM 3928. Phylogenetic inference was performed using MEGA X with 1000 bootstraps and the Jukes Cantor method. *Bacillus licheniformis* t.s. DSM 13 was used as outgroup.

**Figure S.3: Phylogenetic affiliation of isolates from the family Micrococcaceae.**

Maximum Likelihood phylogram of Micrococcaceae based on 16s rDNA. Two isolates with high sequence similarity with *Micrococcus parietis*, *M. muralis* and *M. alkalitolerans* were confirmed as *M. parietis* by phylogenetic association with t.s. CCM 7609. Two isolates were confirmed as *M. luteus*; two as *Kocuria rhizophila*; and three as *Glutamicibacter protophormiae*. Phylogenetic inference was performed using MEGA X with 1000 bootstraps and the Jukes Cantor method. *Bacillus aerius* 24K was used as outgroup.

Phylum Pseudomonadota

Class Alphaproteobacteria

**Figure S.4: Phylogenetic affiliation of isolates from the family Erythrobacteraceae.**

Maximum Likelihood phylogram of Erythrobacteriaceae based on 16s rDNA. (A) Nine *Erythrobacter* isolates with high database sequence similarity with uncultured species previously observed in the deep sea of arctic regions were confirmed as associated to this group of organisms, which form an independent clade closely related to *Erythrobacter citreus*. (B) Six isolates with high database similarity grouped together with *Erythrobacter citreus* t.s. RE35F/1, while two isolates matching *E. citreus* in nBlast grouped in a clade with *E. pelagi* at considerable branch distance and were assigned as *Erythrobacter* sp. (SFI-P2) and *Erythrobacter* sp.1 (SFI-C154). Three species closely associated in database with *E. seohaensis* were taxonomically confirmed. Phylogenetic inference was performed using MEGA X with 1000 bootstraps and the General Time Reversible method.

(A)

(B)

**Figure S.6: Phylogenetic affiliation of isolates from the family Aurantimonadaceae.**

Maximum Likelihood phylogram of Aurantimonadaceae based on 16s rDNA. Two out of three isolates in this family with high database sequence similarity with *Aurantimonas coralicida* were phylogenetically confirmed, while one (SFI-C26) was assigned as *Aurantimonas* sp. as grouped independently with considerable branch distance. Phylogenetic inference was performed using MEGA X with 1000 bootstraps and the General Time Reversible method. *Bacillus licheniformis* t.s. DSM 13 and *B.aerius* 24K were used as outgroup.

**Figure S.5: Phylogenetic affiliation of isolates from the family Hyphomonadaceae.**

Maximum Likelihood phylogram of Hyphomonadaceae based on 16s rDNA. One isolate with multiple database matches showed to clade independently, closely related to *Hyphomonas jannaschiana* DSM 5153 and *H. adhaerens* ATCC 43965, therefore ultimately assigned as *Hyphomonas* sp. Phylogenetic inference was performed using MEGA X with 1000 bootstraps and the General Time Reversible method. *Bacillus licheniformis* t.s. DSM 13 was used as outgroup.

**Figure S.7: Phylogenetic affiliation of isolates from the family Rhodobacteraceae.**

Maximum Likelihood phylogram of Rhodobacteraceae based on 16s rDNA. Six isolates showed high database sequence similarity with unidentified *Sulfitobacter* or *S. pseudonitzschiae*. Four of these isolates grouped together with *S. pontiacus* and were accordingly assigned, while two were assigned as *S. pseudonitzschiae.* One isolate in this family with high database sequence similarity with unidentified *Loktanella, Limimaricola cinnabarinus*, and *Methylarcula* sp. was ultimately assigned as *L. cinnabarinus*. Phylogenetic inference was performed using MEGA X with 1000 bootstraps and the General Time Reversible method. *Bacillus licheniformis* t.s. DSM 13 was used as outgroup.

Phylum Pseudomonadota

Class Gammaproteobacteria

**Figure S.8: Phylogenetic affiliation of isolates from the family Halomonadaceae.**

Maximum Likelihood phylogram of Halomonadaceae based on 16s rDNA. Ten isolates were confirmed as *Halomonas meridiana* by grouping with the t.s. DSM 5425. Six isolates showing high database sequence similarity with *Halomonas venusta* t.s. DSM 4743 were confirmed, and two isolates with high sequence similarity with *H. titanicae* t.s. BH11 were phylogenetically confirmed. Phylogenetic inference was performed using MEGA X with 1000 bootstraps and the Jukes Cantor method. *Bacillus subtilis* was used as outgroup.

**Figure S.9: Phylogenetic affiliation of isolates from the family Alteromonadaceae.**

Maximum Likelihood phylogram of Alteromonadaceae based on 16s rDNA. One isolate (SFI-N15) with high sequence similarity with *Alteromonas undina* was confirmed by phylogenesis, and showed remarkable genetic variation compared to the t.s. NCIMB 2128T. One isolate (SFI-C87) with high sequence similarity with *Pseudoalteromonas issachenkonii* and *P. tetraodonis* grouped together with both and was ultimately assigned as *Pseudoalteromonas sp.* Two isolates (SFI-C2, CFI-C110) with low sequence similarity in Blast nucleotide were ultimately assigned as *Marinobacter* sp. as they grouped in a lineage closely related to a clade including *Marinobacter bryozoorum* and *M. segnicrescens*. Three isolates showed high sequence similarity with *Alteromonas marina*, *A. macleodii*, *A. tagae*, and *A.* naphthalenivorans: one isolate was ultimately assigned as *A. naphthalenivorans*, while the isolates SFI-C86 and SFI-C95 showed affiliation with multiple species and therefore were assigned as *Alteromonas sp.* Phylogenetic inference was performed using MEGA X with 1000 bootstraps and the Jukes Cantor method. *Bacillus licheniformis* t.s. DSM 13 was used as outgroup.

**Figure S.10: Phylogenetic affiliation of isolates from the family Pseudomonadaceae.**

Maximum Likelihood phylogram of Pseudomonadaceae based on 16s rDNA. A group of eight isolates showed high database sequence similarity with *Pseudomonas xanthomarina, P. kunmingensis* and *P. zhaodongensis.* Phylogenetic analysis highlighted five isolates forming two independent clades closely associated with *P. xanthomarina* and *P. kunmingensis,* therefore assigned as *Pseudomonas* sp.1 (SFI-C145, SFI-C96, SFI-P4) and *Pseudomonas* sp.2 (SFI-C88, SFI-C125); and three isolates confirmed as *Pseudomonas zhaodongensis*. Two isolates with high sequence similarity with *P. pachastrallae* and *P. abyssi* were ultimately assigned as *P. abyssi*. Phylogenetic inference was performed using MEGA X with 1000 bootstraps and the Jukes Kantor method. *Bacillus licheniformis* t.s. DSM 13 was used as outgroup.

**Figure S.11: Phylogenetic affiliation of isolates from the family Moraxellaceae.**

Maximum Likelihood phylogram of Moraxellaceae based on 16s rDNA. Six isolates were taxonomically assigned to the genus *Psychrobacter* according to similarity in Blast database. Phylogenesis allowed assignment to species level of four isolates: *P. celer* (SFI-C52)*, P. nivimaris* (SFI-A15)*, P. okhotskensis* (SFI-A16)*, P. pulmonis* (SFI-A12); while two isolates grouped together independently in a branch closely related to *P. pulmonis* and and were assigned as *Psychrobacter* sp. Phylogenetic inference was performed using MEGA X with 1000 bootstraps and the General Time Reversible method. *Moraxella atlantica* t.s. CCUG 6415 was used as outgroup.

Phylum Firmicutes

**Figure S.12: Phylogenetic affiliation of isolates from the family Bacillaceae.**

Maximum Likelihood phylogram of Bacillaceae based on 16s rDNA. (A) A group of 11 isolates with high sequence similarity with *Bacillus licheniformis* and *B. paralicheniformis* were analyzed independently to distinguish affiliation to the two closely related species: 9 isolates were assigned as *B. licheniformis* and two (SFI-C139 and SFI-C140) as *B. paralicheniformis.* (B) Another group of 19 isolates with sequence similarity with multiple species was analyzed. Seventeen isolates were taxonomically assigned according to the phylogenetic match with closely related type strain sequences, while while two isolates remained unidentified: SFI-C5 was taxonomically assigned as *Bacillus* sp.1 and SFI-C10 was assigned as *Bacillus* sp.2. Phylogenetic inference was performed, in both cases, using MEGA X with 1000 bootstraps and the Jukes Kantor method. *Erythrobacter citreus* t.s. RE35F/1 was used as outgroup.

(A)

(B)

Phylum Bacteroidetes

**Figure S.13: Phylogenetic affiliation of isolates from the family Flavobacteriaceae.**

Maximum Likelihood phylogram of Flavobacteriaceae based on 16s rDNA. One isolate with high sequence similarity with *Leeuwenhoekiella* *aequorea* was confirmed, while one isolate of *Gramella* with high sequence similarity with multiple species in database, grouped phylogenetically with *Gramella marina* and was ultimately assigned. Phylogenetic inference was performed using MEGA X with 1000 bootstraps and the General Time Reversible method. *Actibacter sediminis* t.s. JC2129 was used as outgroup.
